# Long-term Double-J stenting is superior to short-term Single-J stenting in kidney transplantation

**DOI:** 10.1371/journal.pone.0317991

**Published:** 2025-01-30

**Authors:** Christiaan A. J. Oudmaijer, Kelly Muller, Erika van Straalen, Robert C. Minnee, Diederik J. A. N. Kimenai, Marlies E. J. Reinders, Jacqueline van de Wetering, Jan N. M. IJzermans, Turkan Terkivatan

**Affiliations:** 1 Division of Hepatobiliary and Transplantation Surgery, Department of Surgery, Erasmus MC Transplant Institute, University Medical Center Rotterdam, Rotterdam, The Netherlands; 2 Division of Nephrology and Transplantation, Department of Internal Medicine, Erasmus MC Transplant Institute, University Medical Center Rotterdam, Rotterdam, The Netherlands; The Fourth Affiliated Hospital Zhejiang University School of Medicine, CHINA

## Abstract

**Background and objectives:**

Urological complications after kidney transplantation, due to the ureteroneocystostomy, are associated with significant morbidity, prolonged hospital stay and even mortality. Ureteral stents can minimize the number of complications but are not consistently used, as previous studies were retrospective in nature. We aim to prospectively determine the most effective stenting approach.

**Methods:**

We performed a non-blinded single-centre randomised controlled trial in an academic hospital. Kidney transplant recipients were randomised to either a Single-J stent or a Double-J stent, removed according to respective protocols. Primary outcome was PCN placement within six months. Secondary outcomes encompassed urinary tract infections, cost-effectiveness, and hospital admission time. The study was conducted from November 2018 to August 2023, during which 300 recipients were included with complete follow-up.

**Results:**

PCN was performed in 14.5% in the Single-J group (21/145) and 4.5% in the Double-J group (7/155), p = 0.003. Multivariable logistic regression, corrected for recipient age, BMI, sex, and donor type, showed an OR of 0.26 [0.10, 0.61] (OR [95%CI]). To prevent PCN in one recipient, 10 would have to receive the Double-J. All secondary outcomes were comparable, whereas hospital admission time and cost-effectiveness analysis heavily favoured Double-J stenting. An important limitation was that Single-J participants were unable to leave, even if their recovery allowed earlier discharge.

**Conclusion:**

This trial showed that Double-J stenting consistently reduced urological complications from 14.5% to 4.5%, while being highly cost-effective. Transplant surgeons should favour Double-J stenting to minimise the risk of complications.

## Introduction

End-stage renal disease (ESRD) is a major cause of morbidity and mortality, necessitating kidney transplantation as a curative treatment [1–4]. Urological complications, such as urinary leakage and ureteral strictures, often necessitate surgical or radiological interventions. Urological complications result in prolonged hospital stays, higher costs, more comorbidities, and rarely even mortality [5,6]. Placement of ureteral stents minimizes the occurrence of urological complications [7,8]. Currently, two types of stents are used: an internalized Double-J stent (which has to be removed via cystoscopy or recently via a magnetized catheter [9,10]) and an externalized Single-J stent which can be removed without additional procedures.

At our centre, we have used the externalized stent for several years, but urological complications have been reported in up to 20% of kidney transplant recipients [11]. Existing literature suggests that the use of Double-J stents may further reduce the incidence of such complications. A retrospective study comprising 76 patients with 43 externalized stents and 33 Double-J stents reported a leakage incidence of 13.9% in the Single-J stent group, compared with 0% in the Double-J stent group [12,13]. Furthermore, the study revealed a significant reduction in hospital stay by 2 days with the use of internal stents [13]. Another retrospective study reviewed the outcomes of 2061 kidney transplant recipients with Double-J, Single-J, and or no stents [14]. Urological complications were observed in 17.3% of the external stent group, 8.4% of the non-stent group, and 5.4% of the Double-J stent group [14]. Another study achieved a reduction in urological complications by transitioning from a non-stented (7.7%) to a Double-J stented (3.8%) ureteroneocystostomy technique [15].

A Cochrane review from 2013, consisting of 7 RCTs of low or moderate quality, showed that routine prophylactic stenting reduced the incidence of major urological complications [16], while recommending additional trials to address the unresolved quality of life and economic issues, simultaneously addressing the low quality of the included studies. All studies in this review had a retrospective design, implicating a significant risk of bias and confounding, thereby limiting the strength and robustness of their findings. Therefore, in our study, we aim to provide conclusive evidence by prospectively evaluating whether Double-J stenting is superior to Single-J stenting in reducing the number of urological complications after kidney transplantation. By conducting this study, we aim to determine the most effective stenting approach, thereby reducing urological complications after transplantation, while simultaneously addressing the unresolved questions regarding quality of life and cost-effectiveness.

## Materials and methods

### Study design

We performed a single-centre randomised controlled trial with a superiority design: the DUET-Trial [17]. The study included patients scheduled for kidney transplantation at the Erasmus MC Transplant Institute, where approximately 200 procedures are performed annually [18]. The intended recipients were approached at the outpatient clinic of the department of surgery during their preoperative screening. Participants were excluded for participation if they had a reconstructed urinary tract, bladder dysfunction that required continuous or intermittent catheterization, were unable to sufficiently understand the Dutch language, and/or received a donor kidney with more than one ureter. A separate special exclusion criterion was primary Focal Segmental GlomeruloSclerosis (FSGS), as the enhanced risk of quick recurrence and kidney dysfunction, including proteinuria, necessitated a Single-J stent [19]. Potential participants were given time to reflect, and when their surgery was scheduled, were asked to provide written informed consent.

The medical ethical committee of Erasmus MC has approved the study protocol, patient information files, consent procedures, and other study related documents and procedures. This medical committee is an extension of the CCMO. The trial has been registered under medical ethical assessment numbers MEC-2016-678 and NL59551.078.16. We prospectively registered this trial and its protocol [17,20].

### Randomisation and intervention

Randomisation was performed after intubation in the operation room, stratified for the type of donor (living/postmortal). Because patients and physicians could notice the presence of an externalized stent postoperatively, the study could not be blinded. Assessment of the outcome and statistical analysis was performed in blinded fashion. Participants who were randomised to Single-J stenting received an externalized 7 French ureteric stent (Teleflex®). Participants who were randomised to Double-J stenting received a short (12 cm) internal Double-J 7 French stent (Teleflex®), with both stents being CE approved and used worldwide. The tip of both stents was positioned in the pelvis of the transplanted kidney. The position of the stent was verified during ultrasonography, which was performed as standard postoperative care the day after surgery.

Recipients were discharged when kidney function was significantly improved or consistently improving, no stents or catheters were in place, and sufficient care was available at home. Transurethral catheters were routinely removed on day 5 for all recipients. External stents were removed 9 days after surgery, according to protocol to minimize the risk of infection [7,8]. Double-J stents were removed after 3 weeks by cystoscopy in the outpatient clinic of the department of Urology.

Study participants underwent the same preoperative screening and workup as the non-participants opting for kidney transplantation. The surgical technique was an open kidney transplantation. After the vascular anastomosis, the transplant surgeon performed an extra vesical anastomosis as described by Lich-Gregoir [21,22]. A myotomy of 2–3 cm on the anterolateral surface of the bladder dome was performed to expose the mucosa of the bladder, after which a small incision was made in the mucosa. The transplanted ureter was trimmed and spatulated posteriorly. The bladder mucosa was sutured to the ureter with a running absorbable suture. The detrusor muscle was closed over the anastomosis by one or two interrupted absorbable sutures to create a submucosal tunnel with an anti-reflux mechanism.

### Primary outcome

Our primary outcome was percutaneous nephrostomy (PCN) placement within six months after transplantation. We opted for this primary outcome given that an often used measure, major urological complications [23], was non-specific for our study subject. PCN placement was performed for either hydronephrosis (defined as dilatation of the renal collecting system of the transplanted kidney) or leakage of urine, both confirmed on abdominal ultrasound and in combination with a decrease in kidney function. We further investigated our primary outcome with logistical regression analysis, a survival analysis including Log-Rank test and we estimated the Number Needed to Treat (NNT). The NNT was calculated as the inverse of the Absolute Risk Reduction (ARR).

### Secondary outcomes

Our secondary endpoints consisted of incidence of catheter dysfunction (defined as either pain, displacement, leakage, not having any urine output, or hydronephrosis without effect on the kidney function), haematuria, urinary tract infection (UTI), urosepsis, reoperation, radiologic interventions, tacrolimus toxicity, kidney replacement therapy, rejection, graft failure, and cost-efficiency. Relevant clinical data were collected from the electronic health record or Eurotransplant. Additionally, recipients were asked to fill out health-related quality of life questionnaires (SF-36 [24]) and the EuroQol (EQ-5D-3L [25]), assessing subjective postoperative recovery: the ability to perform daily tasks, health outcome and physical and mental health [26–28]. Questionnaires were completed at baseline, 2 & 6 weeks, and 6 months after transplantation. The cost effectiveness of the Double-J stent was estimated using internal billing codes. The costs of the Double-J stent included the costs of cystoscopy and personnel in the outpatient clinic. Costs for PCNs were included using the percentage of PCNs multiplied by the costs. Primary admittance costs, for both the Single-J and Double-J, were included using the mean days admitted per group.

### Statistical analysis

Based on a two-sided alpha of 0.05 and a β of 0.8, we calculated that 149 patients were needed per trial arm to reject the null hypothesis of no effect [17]. We estimated that the Double-J stent would reduce the probability of PCN placement from 9% to 1.5% [8,11]. Statistical analysis was performed using R version 4.0.3 or higher. A two-sided significance level of 0.05 was used for all primary and secondary analyses, unless otherwise stated. Statistical tests (t-test, Chi-square, and Wald-test) were performed where applicable, depending on the type of variable. Clinical outcomes, such as the placement of PCN, incidence of delayed graft function (DGF) and acute rejection (AR), were calculated and compared as proportions.

Statistical models were built according to current standards [29] to determine the effect of the intervention on the outcome, adjusting for relevant factors such as age, sex, and recipient of a living or deceased donor kidney. Specifically, for the effect of the intervention on the primary outcome, we adjusted the logistic regression model for the type of donor (living/postmortal), recipient sex, age, and BMI. These factors were deemed clinically relevant, given that they affect the vitality of the surgical anastomosis and therefore our outcome. In addition, a survival analysis using the Kaplan-Meier method was employed to correct for possible censoring of the primary endpoint due to recipient death in the first 6 months of follow-up. Regression analysis assumptions, including linearity, homoscedasticity, and normality, were visually checked. Statistical analysis for secondary endpoints was corrected for multiple testing using the Bonferroni method.

## Results

### Screening and inclusion

**Fig 1:**
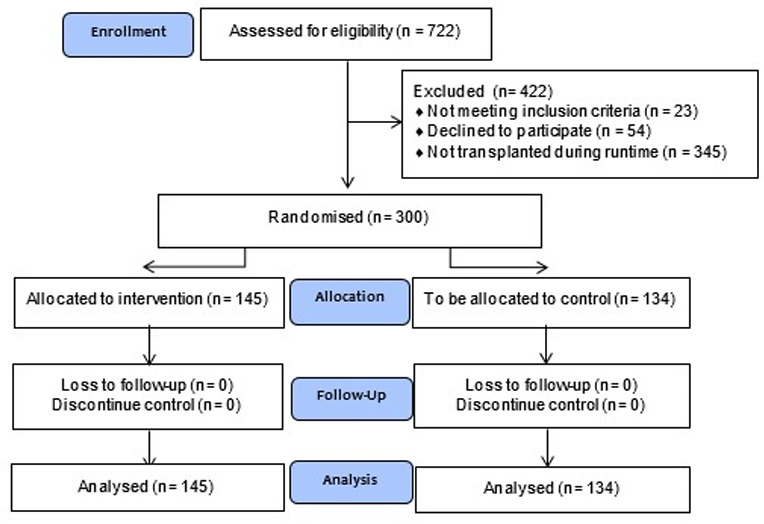
Flowchart of study inclusion and randomization. Legend: Flowchart providing visual information on study participants inclusion and retention.

Inclusion and study participation ran from November 2018 to August 2023, during which 300 recipients were included. In total, 722 participants were screened for eligibility. Twenty-three participants were excluded because of their urological history and/or FSGS as cause of their renal disease, and the remaining 699 patients were informed. Out of these 699 eligible patients, 300 participants were included, 54 declined, and the remaining 345 participants were not transplanted during the study runtime, as they were still on the waiting list for a deceased donor, had not been transplanted yet, or were transplanted during the Covid19 pandemic, during which the trial was not active due to hospital regulations. Fig 1 represent the flowchart of our randomised trial; 145 Participants received the Single-J and 155 received the Double-J. Randomisation resulted in two groups with no baseline differences, for both recipients and donors, with the results presented in Tables 1 and 2.

**Table 1:** Donor baseline characteristics.

	Single J (N = 145)	Double J (N = 155)	Total (N = 300)
**Donor type**			
Deceased	64 (44.1%)	69 (44.5%)	133 (44.3%)
Living	81 (55.9%)	86 (55.5%)	167 (55.7%)
**Donor Age**			
Mean (SD)	56.9 (13.9)	54.8 (14.3)	55.8 (14.1)
Range	11–80	5–81	5–81
**Donor Sex**			
Female	80 (55.2%)	70 (45.2%)	150 (50.0%)
Male	65 (44.8%)	85 (54.8%)	150 (50.0%)
**Donor BMI**			
Mean (SD)	26.5 (4.4)	26.5 (4.1)	26.5 (4.3)
Range	17.7–44.4	12.1–40.0	12.1–44.4
**Donor ASA**			
I	36 (44.4%)	26 (30.2%)	62 (37.1%)
II	42 (51.9%)	57 (66.3%)	99 (59.3%)
III	3 (3.7%)	3 (3.5%)	6 (3.6%)
**Donor Smoking**			
Missing	2	3	5
Never	63 (44.1%)	65 (42.8%)	128 (43.4%)
In the past	44 (30.8%)	38 (25.0%)	82 (27.8%)
Active smoker	36 (25.2%)	49 (32.2%)	85 (28.8%)
**DCD or DBD**			
DCD	41 (64.1%)	40 (58.0%)	81 (60.9%)
DBD	23 (35.9%)	29 (42.0%)	52 (39.1%)
**Side donated**			
Left	92 (63.4%)	98 (63.2%)	190 (63.3%)
Right	53 (36.6%)	57 (36.8%)	110 (36.7%)
**Surgical technique**			
Laparoscopic	78 (96.3%)	78 (90.7%)	156 (93.4%)
Hand-assisted	2 (2.5%)	3 (3.5%)	5 (3.0%)
Robot-Assisted	1 (1.2%)	5 (5.8%)	6 (3.6%)

Legend: SD = Standard Deviation, ASA = American Society of Anaesthesiologists, BMI = Body Mass Index, DCD = Deceased after cardiac death, DBD = Deceased after brain death.

**Table 2:** Recipient baseline characteristics.

	Single J (N = 145)	Double J (N = 155)	Total (N = 300)
**Recipient Sex**			
Female	63 (43.4%)	52 (33.5%)	115 (38.3%)
Male	82 (56.6%)	103 (66.5%)	185 (61.7%)
**Recipient Age**			
Mean (SD)	59.9 (13.0)	58.3 (13.5)	59.1 (13.3)
Range	23.5–83.4	22.3–83.3	22.3–83.4
**Recipient BMI**			
Mean (SD)	27.5 (5.4)	27.4 (5.1)	27.5 (5.3)
Range	13.6–43.6	17.6–41.2	13.6–43.6
**Recipient ASA**			
II	15 (10.3%)	11 (7.1%)	26 (8.7%)
III	112 (77.2%)	122 (78.7%)	234 (78.0%)
IV	18 (12.4%)	22 (14.2%)	40 (13.3%)
**Recipient Smoking**			
Never	66 (45.5%)	59 (38.1%)	125 (41.7%)
In the past	59 (40.7%)	74 (47.7%)	133 (44.3%)
Active smoker	20 (13.8%)	22 (14.2%)	42 (14.0%)
**Pre-emptive Surgery**			
Not Pre-emptive	86 (59.3%)	103 (66.5%)	189 (63.0%)
Pre-emptive	59 (40.7%)	52 (33.5%)	111 (37.0%)

Legend: SD = Standard Deviation, ASA = American Society of Anaesthesiologists, BMI = Body Mass Index.

### Primary outcome

The primary study endpoint, PCN placement within six months after transplantation, was significantly reduced in the Double-J group (N = 7, 4.5%), compared with the Single-J (N = 21, 14.5%), p = 0.003. In addition to the Chi-Square test, unadjusted logistic regression analysis resulted in an OR of 0.28 (95% CI [0.11:0.65], p = 0.005) for the Double-J. When corrected for sex, type of donor, age, and BMI, regression analysis resulted in an OR of 0.26 (95% CI [0.10:0.61], p = 0.003). After reviewing their statistical relevance [29], only BMI remained a statistically significant confounder. In the logistic regression analysis adjusted for only BMI, Double-J had an OR of 0.28 (95% CI [0.11:0.65], p = 0.005), and a higher BMI was non-significantly associated with a higher chance of PCN (OR = 1.01, 95% CI [0.94:1.09], p = 0.71). The number needed to treat with Double-J was estimated at 10; to prevent PCN placement in one recipient, 10 would have to receive the Double-J.

**Fig 2:**
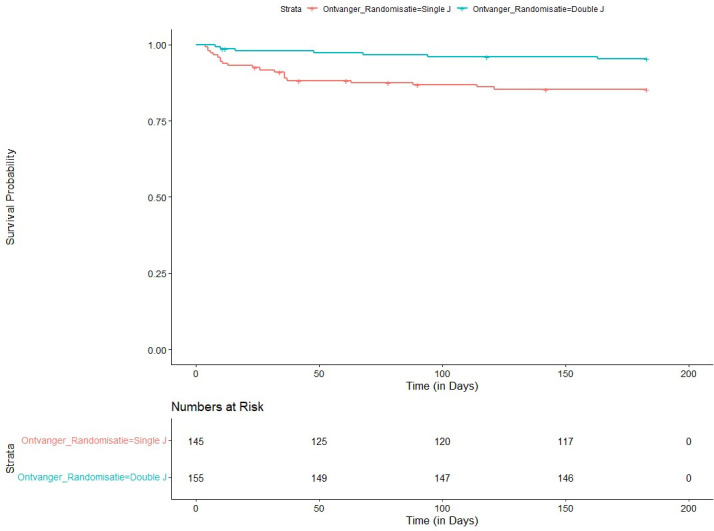
Kaplan-Meier curve for the primary outcome. Legend: Graph estimated via the Kaplan-Meier method, displaying the numbers at risk for PCN, occurrence and timing of events (PCN) for both study arms. PCNs in the Double-J and Single-J groups occurred over the entire follow-up period, but the Single-J group showed markedly more in the first 2–3 weeks.

The mean time to PCN was 33 days in the Single-J, compared with 58 days in the Double-J. During study runtime, 3 (1.9%) patients in the Double-J died, compared to 8 (5.5%, p = 0.099) in the Single-J arm. To account for censoring due to death, we investigated the time to PCN via survival analysis, as shown in Fig 2. PCNs in the Double-J and Single-J groups occurred over the entire follow-up period, but the Single-J group showed markedly more in the first 2–3 weeks. When analysing this via the Log Rank test, Double-J was statistically superior (p = 0.003). When discerning the reason for PCN in the Single-J-Group, it was mostly due to hydronephrosis of the transplanted kidney (n = 16, 76.2%) or leakage of urine (n = 5, 23.8%). In the Double-J, this was in 4 cases due to hydronephrosis (57.1%) and in 3 cases due to leakage (42.9%).

### Secondary outcomes

#### Stent/Catheter dysfunction and clinical outcomes.

Dysfunction of the catheter was highly prevalent in the Single-J arm (n = 48, 33.3%), compared with Double-J (n = 9, 5.8%), which was statistically significant (p < 0.001). The other secondary outcomes revealed no substantial disparities between the groups, with all p-values larger than 0.25, see also S1 Table. 65.0% of the cohort did not experience UTI, with comparable rates between Single-J (62.1%) and Double-J (67.7%). Urosepsis occurred in 8.7% of all cases, with no significant differences noted. Haematuria was observed in 24.7% of cases, with similar rates in the Single-J (24.8%) and Double-J (24.5%) groups. Radiologic intervention was required in some cases; four across the total cohort underwent dilatation of a stenosis, with no significant difference between stent types. Tacrolimus toxicity was noted in 56.7% of cases, with no significant difference between Single-J (55.9%) and Double-J (57.5%) groups. Postoperative dialysis was required in 22.3% of cases, with no significant difference between the two groups. Methylprednisolone for a suspected graft rejection was given in 24.3% of all cases, with comparable rates between Single-J (26.9%) and Double-J (21.9%) groups. Acute rejection was proven in a biopsy in 37 participants (12.3%), which was again comparable across both groups. Of all participants, 285 (95.0%), did not experience graft failure during the first 6 months after transplantation.

#### Surgical outcomes.

When comparing the time of surgery, warm ischemia time, cold ischemia time, and length of hospital stay, we initially found that the time of surgery was significantly shorter in the Single-J group (116 vs 126 minutes), p-value: 0.029. No significant differences were observed in the cold or warm ischemia time. The length of hospital stay differed significantly, with Single-J noting an average of 12.7 days and Double-J 9.6 days, p < 0.001. After correcting for multiple testing, the mean surgery time, and cold and warm ischemia time did not show significant differences. However, the difference in days admitted stayed highly significant (p < 0.001), indicating a robust finding of a longer duration of hospital stay for the Single-J compared to the Double-J, see also S2 and S3 Tables. Additionally, when comparing whether participants were readmitted in the first 6 months after transplantation, we that in the Single-J group, 64 (44.1%) recipients were readmitted, while in the Double-J group 67 (43.2%) recipients were readmitted (p = ns).

#### Postoperative recovery of kidney function.

Regarding the postoperative recovery of kidney function, we did not find any statistically or significant effect. At the end of the first week, mean creatinine levels were 228 for Single-J and 265 μmol/L for Double-J, with a non-significant p-value of 0.197. The eGFR showed similar trajectories for both groups, 36 for Single-J and 37 ml/min for Double-J. At week 4, mean creatinine levels were 165 for Single-J and 170 μmol/L for Double-J, with an eGFR of 43 & 45 ml/min, respectively. By week 12, the mean creatinine levels were 156 for Single-J and 164 μmol/L for Double-J, with the eGFR estimated at 44 and 46 ml/min, respectively. At the end of the study runtime, mean creatinine levels were 150 for Single-J and 153 μmol/L for Double-J, with the eGFR estimated at 47 & 48 ml/min, respectively. These findings are reported in S4 Table and S1 and S2 Figs.

#### Patient-centred outcomes.

Regarding the patient-centred outcomes, we found that the PCS score was statistically significantly better at the 6- and 26-weeks mark for the Double-J group. When corrected for residual differences at baseline, Double-J resulted in an improvement of 2.4 points (p = 0.031) at 6 weeks and 4.05 points (p = 0.001) at 26 weeks. Additionally, at the 26-week mark, higher age was associated with a lower score (−0.14 per year, p = 0.003) and pre-emptive transplantation was associated with a higher score (3.0 points, p = 0.017). MCS score was comparable across both groups at the three time points, as illustrated in S3 and S4 Figs. When studying the sub-scores at 2 weeks, we found that both Physical Functioning and Mental Health were significantly improved in the Double-J group, 8.0 points (p = 0.029) and 4.7 points (p = 0.037), respectively. Both analyses were corrected for baseline score, age, and sex. At 6 weeks, all these subdomains were comparable between the two study arms. At the study endpoint, the domains of Physical Functioning, Vitality and General Health scored higher in the Double-J arm: 8.6 (p = 0.018), 6.8 (p = 0.036) and 5.5 (p = 0.034), respectively, corrected for age, sex, pre-emptive transplantation, and baseline score. See S5 and S6 Figs for the trajectories after surgery. The EQ-5D-5L Index and VAS scores showed similar postoperative trajectories for both groups. The index score showed a similar postoperative reduction, after which the score returned to preoperative levels during follow-up. The self-rated VAS showed a clear increase after surgery, comparing every time point with baseline resulted in; t: 5.6, p < 0.001, t: 6.7, p < 0.001 and t: 7.4, p < 0.001, chronologically.

#### Costs of treatment.

When analysing the costs of treatment, costs of a day of admittance on the transplantation ward was estimated at €725.52, and the placement of PCN at €857.80 (including the radiologist, the procedure itself and necessary material). It is important to note that we did include the costs of therapy after PCN placement, as this was highly variable. The costs of the Double-J placement and removal (including outpatient clinic cost of both the urologist and cystoscopy) were estimated at €223.57. The price of placement and removal of Single-J was noted at € 50.36, as removal was done during the primary admittance by a certified nurse. When including costs of primary hospital stay, Double-J costs were estimated at € 6936.91 and Single-J costs at € 9191.59. Without including any re-admittance fees or prices of PCN, we estimated that the Single-J was €2081.47 costlier. Taking the risk and price of PCN into account, but not the admittance fees, we found that the Single-J was € 2167.25 more expensive. PCN placement could both be performed during primary admittance, during a day admittance after diagnosis at the outpatient Nephrology clinic, or during a new clinical (re-)admittance. When adding these admittance fees, we found that Single-J was € 4574.76 costlier. Most likely, the costs of the Single-J are still an underestimation, given we did not include the costs due to therapy for the stenosis or leakage itself, being either radiological or surgical intervention, as this was highly variable.

## Discussion

In our randomised controlled trial, we found a significant reduction in urological complications, indicating a consistent advantage of the Double-J. Clinically relevant secondary outcomes revealed no substantial differences between the two arms. Markedly, we did find that patients with a Double-J could be safely discharged earlier, reducing the burden of care. This finding is not due to the stent per se, but is the result of the stenting protocol, with the Single-J removed on day 9 compared to the Double-J placement for three weeks. It is important to note that the Double-J facilitates a safe and earlier discharge home. Cost-effectiveness estimations favoured the Double-J, and is likely still an underestimation, as we did not include the cost of therapy of urological complications. Postoperative kidney function, assessed through creatinine levels and eGFR, did not demonstrate statistically significant differences between the study groups. Subjective outcomes were mostly comparable, but the Double-J group showed improved PCS scores at specific postoperative time points when compared with the Single-J arm, displaying potential advantages in physical wellbeing, while several subdomains also showed improved scores.

Interestingly, we found that the incidence of PCN in the Single-J arm (14.5%) was higher than estimated before in a previous trial conducted at our centre (8%, [30]). We hypothesize that this difference in urological complications is due to the aging and more complex patient group currently opting for kidney transplantation. An important limitation of our study was that participants with the Single-J stent were unable to leave the hospital because of this of type stent, even if their recovery allowed earlier discharge. This is due to the risk carried by inadequate care of the stent, and the additional patient burden of returning to the hospital 2–3 days later for removal. However, because this RCT was designed as a pragmatic RCT comparing the implementation of both procedures, we conclude that this finding has important implications for clinical care. Stenting with a Double-J facilitates early discharge, while having no adverse effects, reducing costs and burden for both healthcare providers and recipients.

Another potential limitation is the long accrual time of 4.5 years, almost exclusively due to the Covid19 pandemic due to hospital regulations and experienced shortage in operating rooms. Another potential concern is the number of participants informed compared with the number that was included, but this is easily explained by study design; when patients were screened at the outpatient clinic, they were informed at that moment to facilitate sufficient time to consider study participation. Consequently, due to the waiting list for either a deceased kidney or surgery schedule, a high number of patients were informed compared to the percentage included, as inclusion was performed at the moment of admittance. This a necessary procedure to facilitate informed decision-making with no risk of selection bias.

As the use of Double-J stents is already widespread, the current study provides novel value by conforming the superiority of this type of stent and facilitates it routine use. It is the first prospective randomised controlled trial providing clear causal evidence, clearly distinguishing itself from the previous retrospective studies. Implications of this study can be profound: the use of a Single-J stent should be limited to select individual cases, such as FSGS. Recently, stents that can be removed by simple catheterization using a magnetized catheter have been developed [9,10]. This could make cystoscopy performed at the outpatient urological department obsolete. While cystoscopy is a relatively simple and quick procedure, alleviating this procedure could further reduce the burden on the patient and the outpatient clinical department, while also cutting costs.

In conclusion, this trial showed that Double-J stenting consistently reduced urological complications while being highly cost-effective. Transplant surgeons should favour Double-J stenting to minimise the risk of complications related to kidney transplantation. As our findings are based on single-center data and experience, future studies could validate our findings in a larger and/or geographically diverse population.

## Supporting information

S1 Table Overview of secondary outcomes. Legend: UTI = Urinary Tract Infection, TT = Tacrolimus Toxicity.
(DOCX)

S2 Table First analysis of study parameters regarding surgery without correction for multiple testing. Legend: SD = Standard Deviation, CI = Confidence Interval.
(DOCX)

S3 Table Second analysis of study parameters regarding surgery with correction for multiple testing. Legend: SD = Standard Deviation, CI = Confidence Interval.
(DOCX)

S4 Table Overview of postoperative creatinine and eGFR. Legend: SD = Standard Deviation, eGFR = estimated glomerular filtration rate. Creatinine was measured in μmol/l, eGFR in ml/min.
(DOCX)

S1 Fig Creatinine at baseline, 2, 6 and 26 weeks after transplantation, for both Single-J and Double-J. (TIFF)

S2 Fig eGFR at baseline, 2, 6 and 26 weeks after transplantation, for both Single-J and Double-J. (TIFF)

S3 Fig Physical Component Score. (TIFF)

S4 Fig Mental Component Score. (TIFF)

S5 Fig Physical Functioning Score trajectory. (TIFF)

S6 Fig Emotional Wellbeing Score trajectory. (TIFF)

S7 Fig Energy Score trajectory. (TIFF)

S8 Fig General Health Score trajectory. (TIFF)

S9 Fig EQ-5D-5L Index-Score trajectory. (TIFF)

S10 Fig EQ-5D-5L VAS-Score trajectory. (TIFF)

S1 Checklist DUET – Consort checklist. (DOCX)

S1 Protocol Study protocol. (PDF)

## Abbreviations

ESRD: End-stage renal disease
VAS: Visual Analog Score
SF-36: 36-Item Short Form Survey
CTA: Computed tomography angiography
DGF: Delayed graft function
AR: Acute rejection
LMM: Linear mixed-effects model
DCD: Deceased after cardiac death
DBD: Deceased after brain death
BMI: Body Mass Index
FSGS: Focal Segmental Glomerulo Sclerosis
PCN: Percutaneous Nephrostomy
DCD: Donation after Circulatory Death
DBD: Donation after Brain Death
NNT: Number Needed to Treat
ARR: Absolute Risk Reduction
PCN: PerCutaneous Nephrostomy.
