# Supplementary material for: Long-term Double-J stenting is superior to short-term Single-J stenting in kidney transplantation
Source: PLoS One. 2025 Jan 30;20(1):e0317991. doi: 10.1371/journal.pone.0317991 (PMC11781732; doi:10.1371/journal.pone.0317991)
Supplement: S1 Table — Legend: UTI = Urinary Tract Infection, TT = Tacrolimus Toxicity. (DOCX) [file pone.0317991.s001.docx]

Supplementary Table 1: overview of secondary outcomes

|  | Single J (N=145) | Double J (N=155) | Total (N=300) | p value (unadjusted) |
| --- | --- | --- | --- | --- |
| **Urinary Tract Infection** |  |  |  | 0.303 |
| No UTI | 90 (62.1%) | 105 (67.7%) | 195 (65.0%) |  |
| UTI | 55 (37.9%) | 50 (32.3%) | 105 (35.0%) |  |
| **Urosepsis** |  |  |  | 0.859 |
| No Urosepis | 132 (91.0%) | 142 (91.6%) | 274 (91.3%) |  |
| Urosepis | 13 (9.0%) | 13 (8.4%) | 26 (8.7%) |  |
| **Haematuria** |  |  |  | 0.950 |
| No Haematuria | 109 (75.2%) | 117 (75.5%) | 226 (75.3%) |  |
| Haematuria | 36 (24.8%) | 38 (24.5%) | 74 (24.7%) |  |
| **Radiologic Intervention** |  |  |  | 0.283 |
| No Radiologic Intervention | 142 (97.9%) | 154 (99.4%) | 296 (98.7%) |  |
| Radiologic Intervention | 3 (2.1%) | 1 (0.6%) | 4 (1.3%) |  |
| **Tacrolimus Toxicity** |  |  |  | 0.786 |
| No TT | 64 (44.1%) | 66 (42.6%) | 130 (43.3%) |  |
| TT | 81 (55.9%) | 89 (57.4%) | 170 (56.7%) |  |
| **Postoperative Dialysis** |  |  |  | 0.509 |
| No need for Dialysis | 115 (79.3%) | 118 (76.1%) | 233 (77.7%) |  |
| Dialysis post-transplantation | 30 (20.7%) | 37 (23.9%) | 67 (22.3%) |  |
| **Solumedrol given** |  |  |  | 0.317 |
| No Solumedrol | 106 (73.1%) | 121 (78.1%) | 227 (75.7%) |  |
| Solumedrol | 39 (26.9%) | 34 (21.9%) | 73 (24.3%) |  |
| **Graft Rejection** |  |  |  | 0.457 |
| No Rejection in biopsy | 125 (86.2%) | 138 (89.0%) | 263 (87.7%) |  |
| Rejection in biopsy | 20 (13.8%) | 17 (11.0%) | 37 (12.3%) |  |
| **Graft Failure** |  |  |  | 0.508 |
| No Graft Failure | 139 (95.9%) | 146 (94.2%) | 285 (95.0%) |  |
| Graft Failure | 6 (4.1%) | 9 (5.8%) | 15 (5.0%) |  |

Legend: UTI = Urinary Tract Infection, TT = Tacrolimus Toxicity
