# Supplementary material for: Long-term Double-J stenting is superior to short-term Single-J stenting in kidney transplantation
Source: PLoS One. 2025 Jan 30;20(1):e0317991. doi: 10.1371/journal.pone.0317991 (PMC11781732; doi:10.1371/journal.pone.0317991)
Supplement: S2 Table — Legend: SD = Standard Deviation, CI = Confidence Interval. (DOCX) [file pone.0317991.s002.docx]

Supplementary Table 2: First analysis of study parameters regarding surgery without correction for multiple testing

|  | Single J (N=145) | Double J (N=155) | Total (N=300) | p value (unadjusted) |
| --- | --- | --- | --- | --- |
| **Surgery Duration**, *in minutes* |  |  |  | 0.029 |
| Mean (SD) | 115.7 (36.8) | 125.5 (41.0) | 120.8 (39.3) |  |
| 95% CI | [109.6, 121.7] | [119.1, 132.1] | [116.3, 125.3] |  |
| Median | 113 | 119 | 115 |  |
| Range | 51- 277 | 45 - 271 | 45 - 277 |  |
| **Cold Ischemia**, *in minutes* |  |  |  | 0.213 |
| Mean (SD) | 358.2 (330.7) | 408.4 (362.4) | 384.1 (347.8) |  |
| 95% CI | [304.0, 412.5] | [350.9, 465.9] | [344.6, 423.7] |  |
| Median | 146 | 154 | 150 |  |
| Range | 71 - 1470 | 76 - 1620 | 71- 1620 |  |
| **Warm Ischemia Total**,  *in minutes* |  |  |  | 0.311 |
| Mean (SD) | 27.2 (11.3) | 28.5 (10.8) | 27.9 (11.1) |  |
| 95% CI | [25.4, 29.1] | [26.8, 30.2] | [26.6, 29.2] |  |
| Median | 24 | 26 | 25 |  |
| Range | 12 - 70 | 11 - 67 | 11 - 70 |  |
| **Warm Ischemia Recipient**,  *in minutes* |  |  |  | 0.034 |
| Mean (SD) | 20.1 (7.5) | 22.9 (8.3) | 21.9 (7.9) |  |
| 95% CI | [19.7, 22.2] | [21.6, 24.2] | [21.0, 22.8] |  |
| Median | 20 | 22 | 20 |  |
| Range | 10 - 53 | 8 - 54 | 8 - 54 |  |
| **Days admittance**, *in days* |  |  |  | < 0.001 |
| Mean (SD) | 12.7 (8.9) | 9.6 (4.8) | 11.1 (7.2) |  |
| 95% CI | [11.2, 14.1] | [8.8, 10.3] | [10.2, 11.9] |  |
| Median | 11 | 8 | 10 |  |
| Range | 7 - 110 | 5 - 37 | 5 - 110 |  |

Legend: SD = Standard Deviation, CI = Confidence Interval.
