# Supplementary material for: Long-term Double-J stenting is superior to short-term Single-J stenting in kidney transplantation
Source: PLoS One. 2025 Jan 30;20(1):e0317991. doi: 10.1371/journal.pone.0317991 (PMC11781732; doi:10.1371/journal.pone.0317991)
Supplement: S4 Table — Legend: SD = Standard Deviation, eGFR = estimated glomerular filtration rate. Creatinine was measured in μmol/l, eGFR in ml/min. (DOCX) [file pone.0317991.s004.docx]

Supplementary Table 4: overview of postoperative creatinine and eGFR

|  | Single J (N=145) | Double J (N=155) | Total (N=300) | p value |
| --- | --- | --- | --- | --- |
| **Week 1: Creatinine** |  |  |  | 0.197 |
| Mean (SD) | 228.4 (191.2) | 264.7 (282.3) | 247.2 (242.9) |  |
| 95% CI | [197.1, 259.8] | [219.9, 309.5] | [219.6, 274.8] |  |
| Median | 154 | 163 | 159 |  |
| Range | 66 - 1544 | 64 - 2437 | 64 - 2437 |  |
| **Week 1: eGFR** |  |  |  | 0.742 |
| Mean (SD) | 34.0 (19.0) | 36.8 (21.84 | 36.4 (20.5) |  |
| 95% CI | [32.9, 39.1] | [33.3, 40.2] | [34.0, 38.7] |  |
| Median | 36 | 38 | 36 |  |
| Range | 3 - 86 | 2 - 88 | 2 - 88 |  |
| **Week 4: Creatinine** |  |  |  | 0.697 |
| Mean (SD) | 164.6 (106.3) | 170.0 (130.7) | 167.4 (119.3) |  |
| 95% CI | [147.2, 182.1] | [149.2, 190.8] | [153.8, 181.0] |  |
| Median | 138 | 137 | 137 |  |
| Range | 67 - 874 | 67 - 1163 | 67 - 1163 |  |
| **Week 4: eGFR** |  |  |  | 0.229 |
| Mean (SD) | 42.8 (16.6) | 45.4 (19.0) | 44.2 (17.9) |  |
| 95% CI | [40.2, 45.6] | [42.4, 48.4] | [42.1, 46.2] |  |
| Median | 42 | 45.500 | 44 |  |
| Range | 5 - 90 | 3 - 108 | 3 - 108 |  |
| **Week 12: Creatinine** |  |  |  | 0.552 |
| Mean (SD) | 155.9 (85.3) | 163.5 (127.0) | 159.8 (108.9) |  |
| 95% CI | [141.7, 170.1] | [143.2, 183.8] | [147.3, 172.3] |  |
| Median | 138 | 131 | 134 |  |
| Range | 65 - 694 | 11 - 1048 | 11 - 1048 |  |
| **Week 12: eGFR** |  |  |  | 0.318 |
| Mean (SD) | 44.7 (16.7) | 46.4 (17.3) | 45.4 (17.0) |  |
| 95% CI | [41.6, 47.2] | [43.6, 49.1] | [43.5, 47.4] |  |
| Median | 43 | 47 | 45 |  |
| Range | 8 - 98 | 3 - 104 | 3 - 104 |  |
| **Week 26: Creatinine** |  |  |  | 0.746 |
| Mean (SD) | 149.4 (91.2) | 152.9 (91.6) | 151.3 (91.3) |  |
| 95% CI | [134.1, 164.8] | [138.2, 167.7] | [140.7, 161.8] |  |
| Median | 130 | 131 | 131 |  |
| Range | 66 - 775 | 72 - 888 | 66 - 888 |  |
| **Week 26: eGFR** |  |  |  | 0.731 |
| Mean (SD) | 47.0 (18.1) | 47.7 (17.7) | 47.3 (17.9) |  |
| 95% CI | [43.9, 50.0] | [44.8, 50.5] | [45.3, 49.4] |  |
| Median | 45 | 47 | 46 |  |
| Range | 6 - 108 | 4 - 101 | 4 - 108 |  |

Legend: SD = Standard Deviation, eGFR = estimated glomerular filtration rate. Creatinine was measured in μmol/l, eGFR in ml/min.
