# Supplementary figures and images for: Long-term Double-J stenting is superior to short-term Single-J stenting in kidney transplantation
Source: PLoS One. 2025 Jan 30;20(1):e0317991. doi: 10.1371/journal.pone.0317991 (PMC11781732; doi:10.1371/journal.pone.0317991)

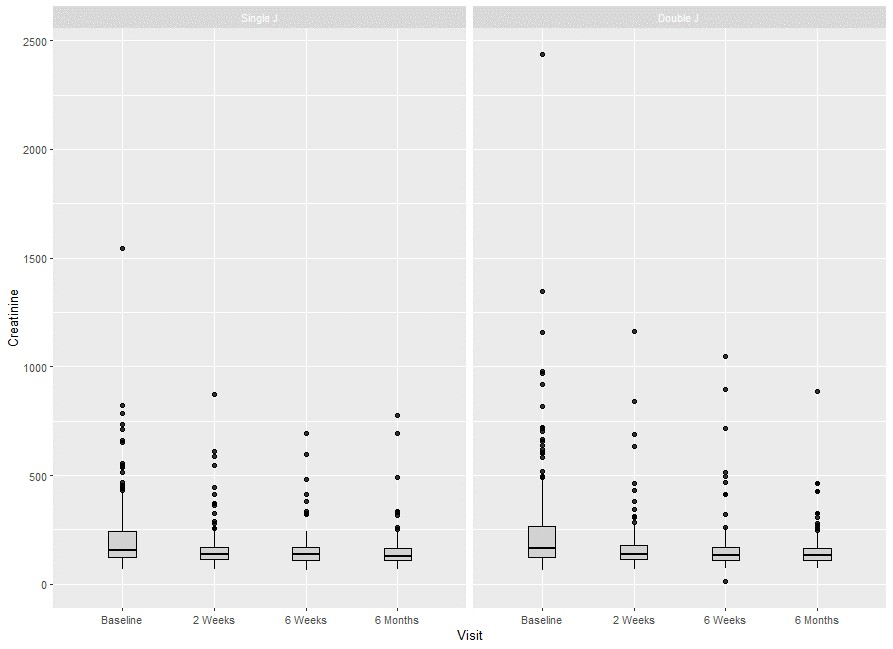

Supplement: S1 Fig — (TIFF) [file pone.0317991.s005.tiff]

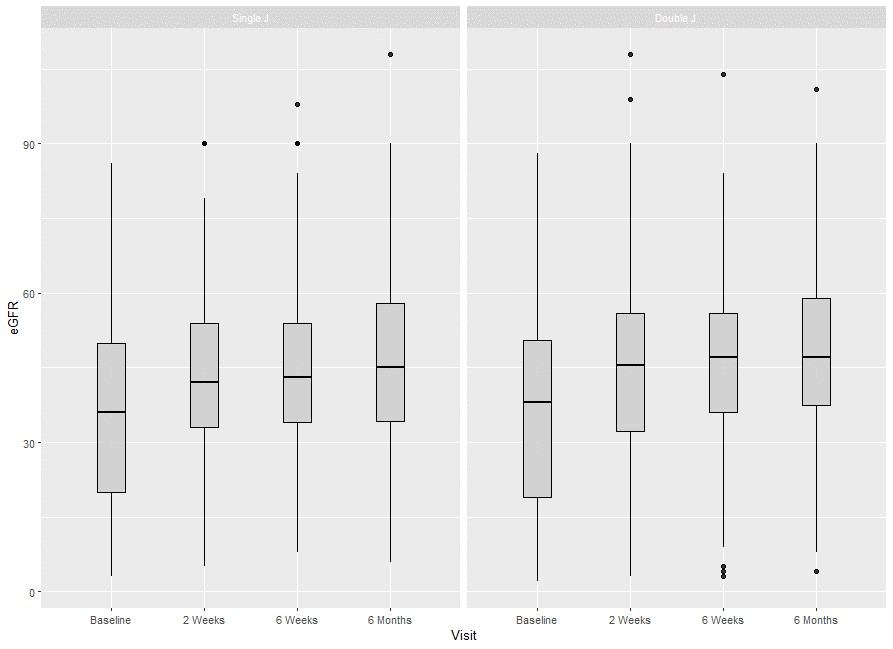

Supplement: S2 Fig — (TIFF) [file pone.0317991.s006.tiff]

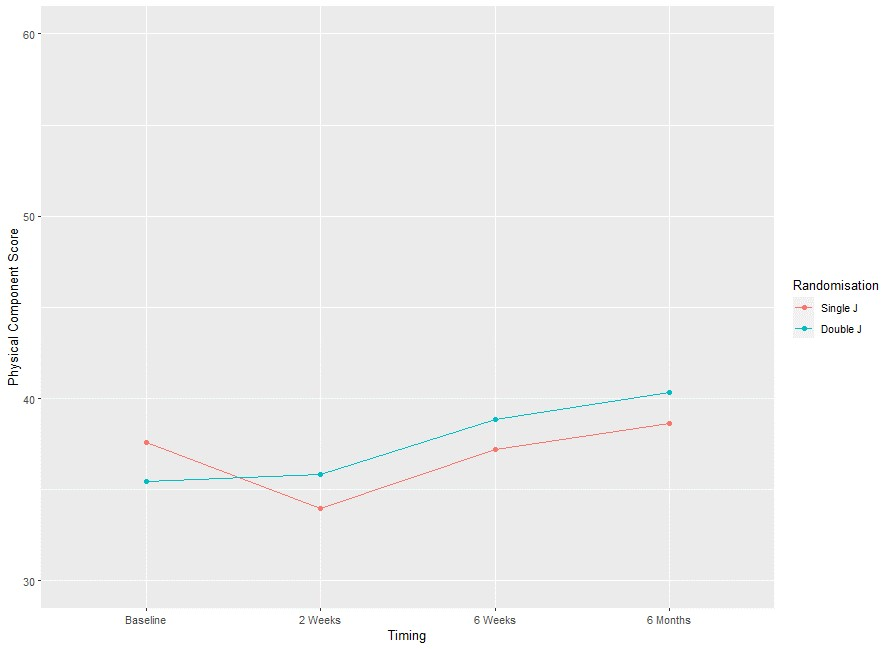

Supplement: S3 Fig — (TIFF) [file pone.0317991.s007.tiff]

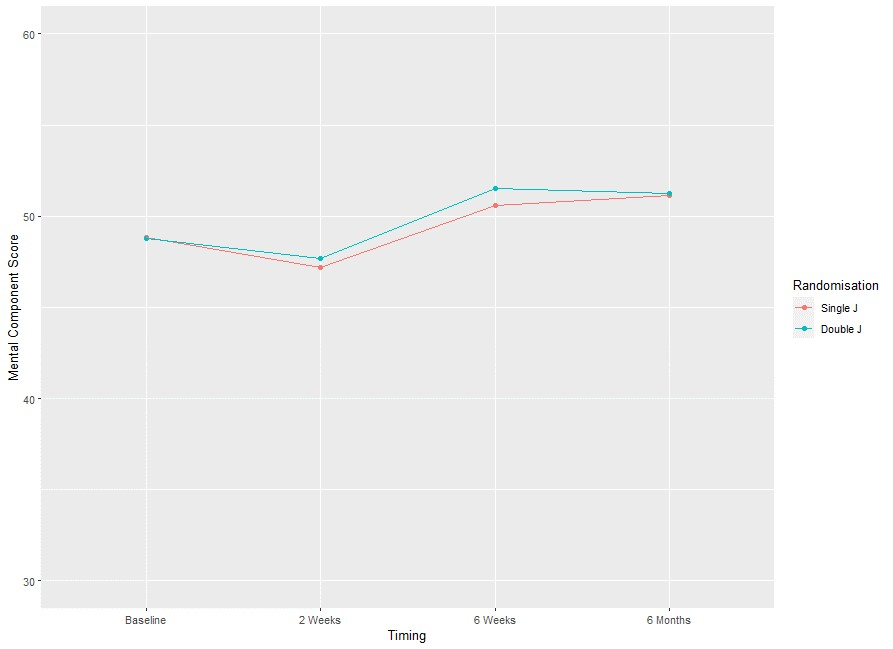

Supplement: S4 Fig — (TIFF) [file pone.0317991.s008.tiff]

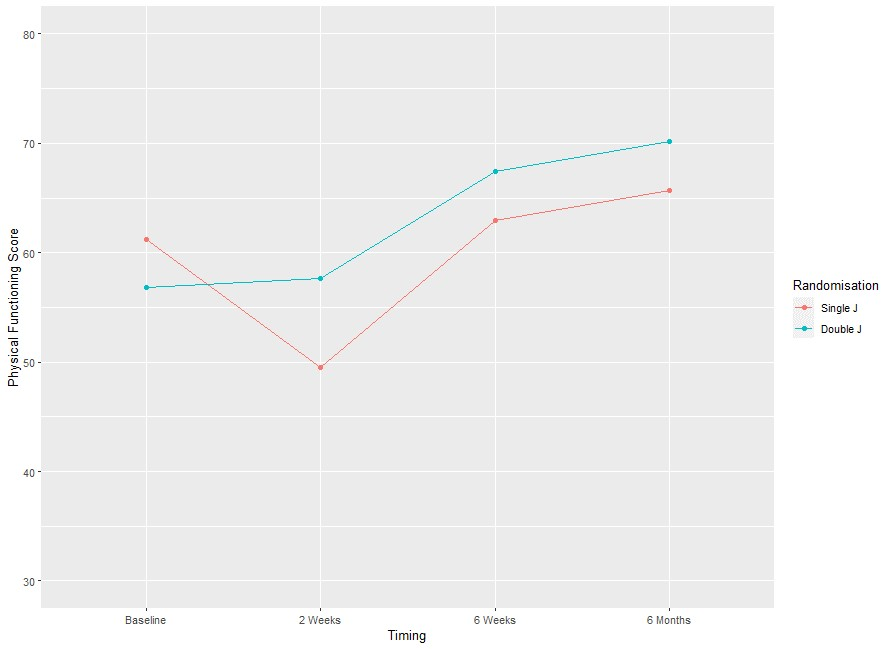

Supplement: S5 Fig — (TIFF) [file pone.0317991.s009.tiff]

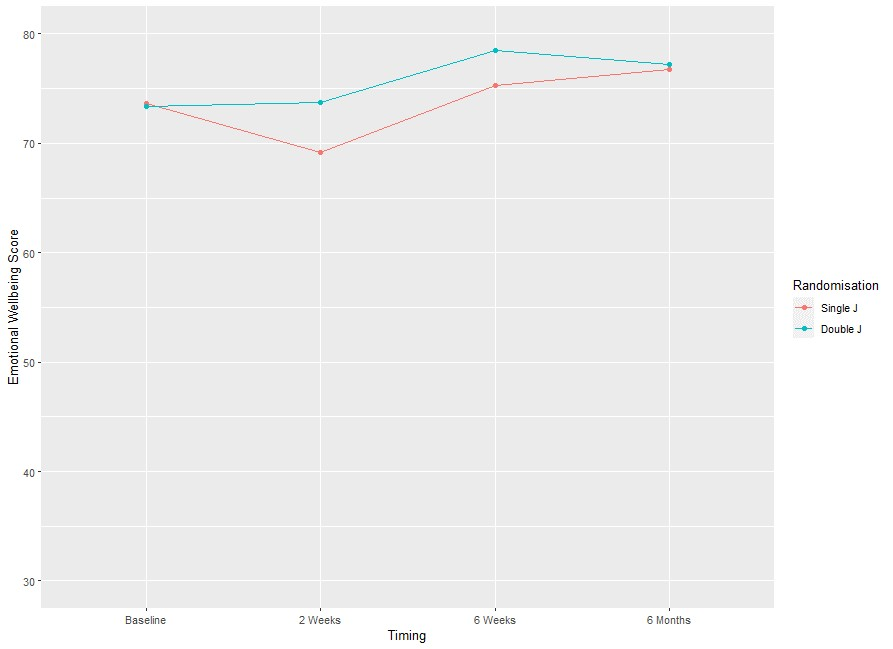

Supplement: S6 Fig — (TIFF) [file pone.0317991.s010.tiff]

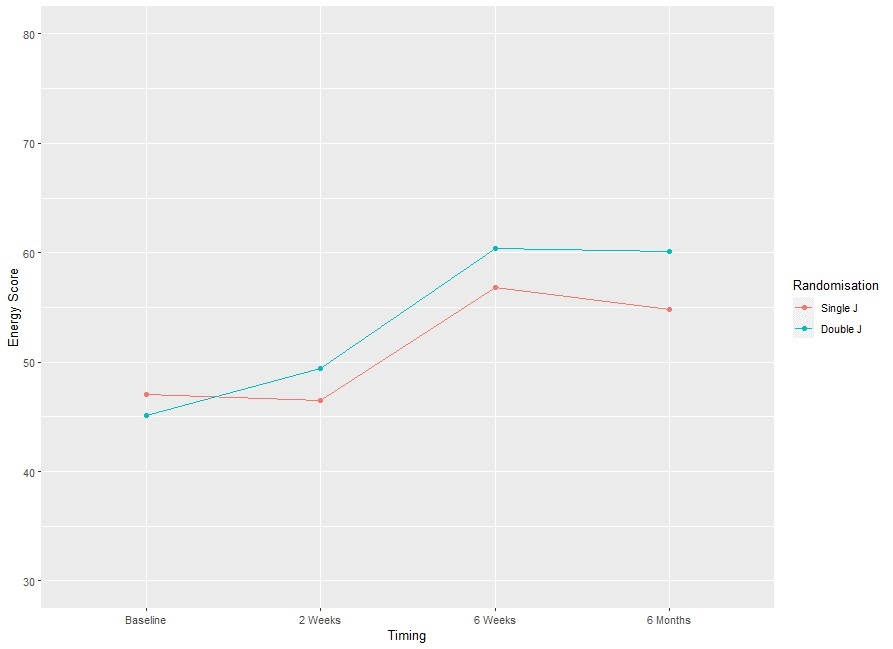

Supplement: S7 Fig — (TIFF) [file pone.0317991.s011.tiff]

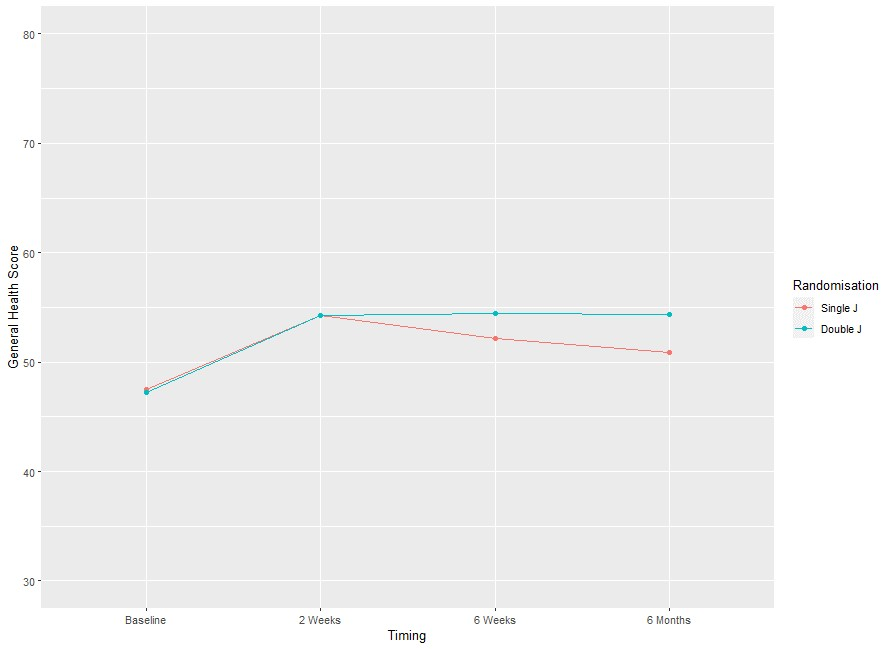

Supplement: S8 Fig — (TIFF) [file pone.0317991.s012.tiff]

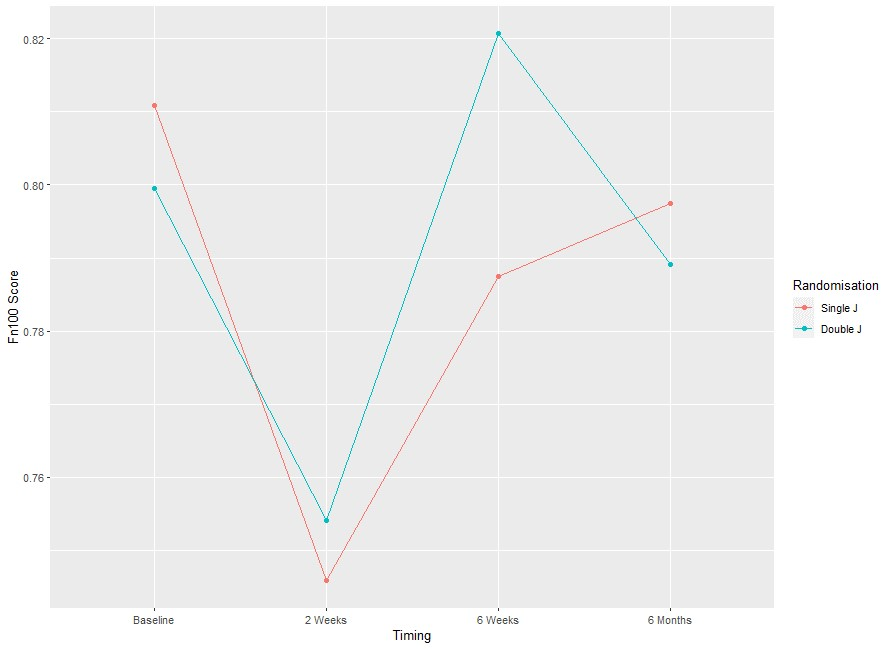

Supplement: S9 Fig — (TIFF) [file pone.0317991.s013.tiff]

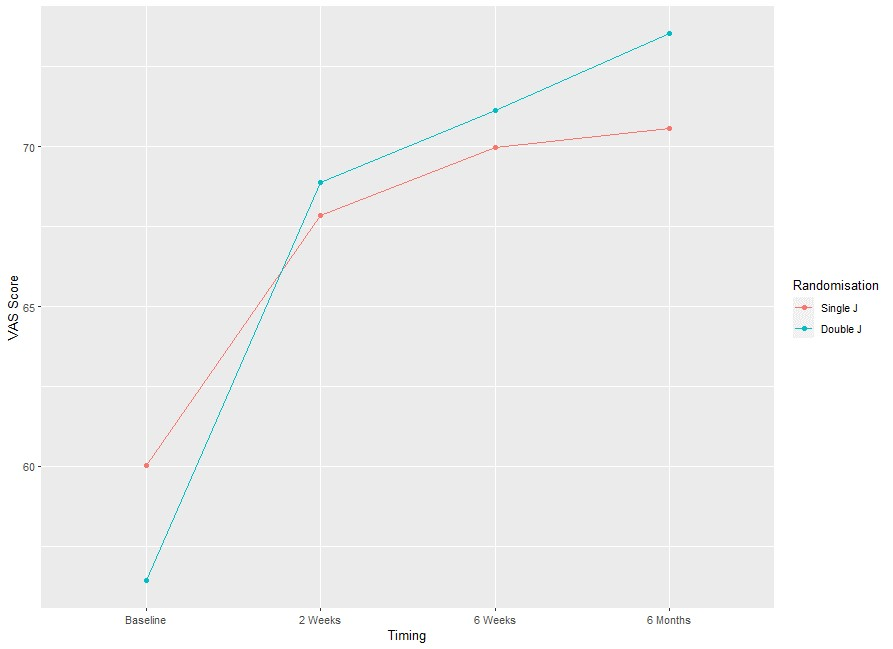

Supplement: S10 Fig — (TIFF) [file pone.0317991.s014.tiff]
