# Supplementary material for: Long-term Double-J stenting is superior to short-term Single-J stenting in kidney transplantation
Source: PLoS One. 2025 Jan 30;20(1):e0317991. doi: 10.1371/journal.pone.0317991 (PMC11781732; doi:10.1371/journal.pone.0317991)
Supplement: S1 Protocol — (PDF) [file pone.0317991.s016.pdf]

DUET-trial; DoUble j or External stenting during kidney Transplantation? Study-protocol

DUET-trial

20-02-2017

Version 2

PROTOCOL TITLE DUET-trial; DoUble j or External stenting during kidney Transplantation? Study-protocol

**Protocol ID**	
**Short title**	**DUET-trial**
**EudraCT number**	***Not applicable***
**Version**	**V2**
**Date**	**20-02-2017**
**Coordinating investigator/project leader:**	***Dr. T. Terkivatan***<br>***T.terkivatan@erasmusmc.nl***
**Principal investigator(s) (in Dutch: hoofdonderzoeker/ uitvoerder)**	***Dr. T. Terkivatan***<br>***T.terkivatan@erasmusmc.nl***
**Sponsor (in Dutch: verrichter/opdrachtgever)**	***Prof. Dr. J.N.M. Ijzermans***<br>***J.ijzermans@erasmusmc.nl***
**Subsidising party**	***Not applicable***
**Independent expert (s)**	***Dr. B.P.L. Wijnhoven***<br>***B.wijnhoven@erasmusmc.nl***
**Laboratory sites <*if applicable*>**	***Not applicable***
**Pharmacy <*if applicable*>**	***Not applicable***

PROTOCOL SIGNATURE SHEET

Name	Signature	Date
**Sponsor or legal representative:**<br>***Prof. J.N.M. IJzermans***<br><br>**Head of Department:**<br>***Prof. J.M. Hendriks***		
**Principal Investigator:**<br>***Dr. T. Terkivatan***		

TABLE OF CONTENTS

1. INTRODUCTION AND RATIONALE 10
2. OBJECTIVES 11
3. STUDY DESIGN 12
4. STUDY POPULATION 13
   - 4.1 Population (base) 13
   - 4.2 Inclusion criteria 13
   - 4.3 Exclusion criteria 13
   - 4.4 Sample size calculation 13
5. TREATMENT OF SUBJECTS 14
   - 5.1 Investigational product/treatment 14
   - 5.2 Use of co-intervention (if applicable) 14
   - 5.3 Escape medication (if applicable) 14
6. INVESTIGATIONAL PRODUCT 15
   - 6.1 Name and description of investigational product(s) 15
   - 6.2 Summary of findings from non-clinical studies 15
   - 6.3 Summary of findings from clinical studies 15
   - 6.4 Summary of known and potential risks and benefits 15
   - 6.5 Description and justification of route of administration and dosage 15
   - 6.6 Dosages, dosage modifications and method of administration 15
   - 6.7 Preparation and labelling of Investigational Medicinal Product 15
   - 6.8 Drug accountability 15
7. NON-INVESTIGATIONAL PRODUCT 16
   - 7.1 Name and description of non-investigational product(s) 16
   - 7.2 Summary of findings from non-clinical studies 16
   - 7.3 Summary of findings from clinical studies 16
   - 7.4 Summary of known and potential risks and benefits 16
   - 7.5 Description and justification of route of administration and dosage 16
   - 7.6 Dosages, dosage modifications and method of administration 16
   - 7.7 Preparation and labelling of Non Investigational Medicinal Product 16
   - 7.8 Drug accountability 16
8. METHODS 17
   - 8.1 Study parameters/endpoints 17
     - 8.1.1 Main study parameter/endpoint 17
     - 8.1.2 Secondary study parameters/endpoints (if applicable) 17
     - 8.1.3 Other study parameters (if applicable) 17

8.2 Randomisation, blinding and treatment allocation 17
8.3 Study procedures 17
8.4 Withdrawal of individual subjects 18
8.4.1 Specific criteria for withdrawal (if applicable) 18
8.5 Replacement of individual subjects after withdrawal 18
8.6 Follow-up of subjects withdrawn from treatment 18
8.7 Premature termination of the study 19
9. SAFETY REPORTING 20
9.1 Temporary halt for reasons of subject safety 20
9.2 AEs, SAEs and SUSARs 20
9.2.1 Adverse events (AEs) 20
9.2.2 Serious adverse events (SAEs) 20
9.2.3 Suspected unexpected serious adverse reactions (SUSARs) 21
9.3 Annual safety report 21
9.4 Follow-up of adverse events 21
9.5 [Data Safety Monitoring Board (DSMB) / Safety Committee] 21
10. STATISTICAL ANALYSIS 23
10.1 Primary study parameter(s) 23
10.2 Secondary study parameter(s) 23
10.3 Other study parameters 234
10.4 Interim analysis (if applicable) 244
11. ETHICAL CONSIDERATIONS 255
11.1 Regulation statement 255
11.2 Recruitment and consent 255
11.3 Objection by minors or incapacitated subjects (if applicable) 25
11.4 Benefits and risks assessment, group relatedness 25
11.5 Compensation for injury 266
11.6 Incentives (if applicable) 26
12. ADMINISTRATIVE ASPECTS, MONITORING AND PUBLICATION 277
12.1 Handling and storage of data and documents 277
12.2 Monitoring and Quality Assurance 27
12.3 Amendments 27
12.4 Annual progress report 27
12.5 End of study report 27
12.6 Public disclosure and publication policy 28
13. STRUCTURED RISK ANALYSIS 299
13.1 Potential issues of concern 29

13.2 Synthesis 29
14. REFERENCES 30

LIST OF ABBREVIATIONS AND RELEVANT DEFINITIONS

**ABR**	**ABR form, General Assessment and Registration form, is the application form that is required for submission to the accredited Ethics Committee (In Dutch, ABR = Algemene Beoordeling en Registratie)**
**AE**	**Adverse Event**
**AR**	**Adverse Reaction**
**CA**	**Competent Authority**
**CCMO**	**Central Committee on Research Involving Human Subjects; in Dutch: Centrale Commissie Mensgebonden Onderzoek**
**CV**	**Curriculum Vitae**
**DSMB**	**Data Safety Monitoring Board**
**EU**	**European Union**
**EudraCT**	**European drug regulatory affairs Clinical Trials**
**GCP**	**Good Clinical Practice**
**IB**	**Investigator's Brochure**
**IC**	**Informed Consent**
**IMP**	**Investigational Medicinal Product**
**IMPD**	**Investigational Medicinal Product Dossier**
**METC**	**Medical research ethics committee (MREC); in Dutch: medisch ethische toetsing commissie (METC)**
**(S)AE**	**(Serious) Adverse Event**
**SPC**	**Summary of Product Characteristics (in Dutch: officiële productinfomatie IB1-tekst)**
**Sponsor**	**The sponsor is the party that commissions the organisation or performance of the research, for example a pharmaceutical company, academic hospital, scientific organisation or investigator. A party that provides funding for a study but does not commission it is not regarded as the sponsor, but referred to as a subsidising party.**
**SUSAR**	**Suspected Unexpected Serious Adverse Reaction**
**Wbp**	**Personal Data Protection Act (in Dutch: Wet Bescherming Persoonsgevens)**
**WMO**	**Medical Research Involving Human Subjects Act (in Dutch: Wet Medisch-wetenschappelijk Onderzoek met Mensen**

SUMMARY

Rationale: Kidney transplantation is the best treatment for patients with chronic kidney failure. Urological complications after kidney transplantation, such as urinary leakage and ureteral strictures, are associated with significant morbidity, surgical and radiological interventions, prolonged hospital stay and even mortality. The majority of urological complications are related to the ureteroneocystostomy and the first treatment for leakage or stenosis of the anastomosis, is placement of a percutaneous nephrostomy (PCN) drain. It has been demonstrated that stent placement can minimize the number of urological complications comparing. Two types of ureteral stents can be used; an internalized double J stent and an externalized single J stent. In our center we have used an externalized stent single J stent for several years and a 9% percentage of urological complications is reported at our center. However, in literature the double J stent has been reported to have a better outcome with less urological complications of 0-5.4%. Unfortunately all these studies are retrospective and no well-designed prospective randomized controlled trials are available on this matter. Therefore, in this study we will investigate whether internal double J stenting is superior to the use of an externalized single J stent in reducing the number of urological complications after kidney transplantation.

Objective: To assess if double J stenting of the ureteroneocystostomy during kidney transplantation is superior in preventing urological complications compared to externalized single J stenting.

Study design: This will be a single-centre randomized controlled trial. Randomization will be performed after intubation in the operation room.

Study population: All adult kidney transplant recipients (>18yrs) are invited to participate. In 3 years 300 recipients will be included. Participants have to understand the Dutch language to sign the informed consent forms and to fill in the questionnaires. Patients will be excluded if they have a reconstructed urinary tract or conduit after total or partial cystectomy, a bladder dysfunction that requires continuous or intermittent catheterization, or will receive a donor kidney with more than one ureter. In addition, patients with primary FSGS and residual urine production will be excluded as well.

Intervention (if applicable): Nowadays, our clinical practice is the use of an externalized single J stent during kidney transplantations. Our “intervention” will be the use of an internal double J stent which will be removed by cystoscopy 3 weeks after transplantation. Alongside, we will ask the recipients to fill in questionnaires to analyse the quality of life and make a cost-effectiveness analysis.

Main study parameters/endpoints: Percutaneous nephrostomy placement.

Nature and extent of the burden and risks associated with participation, benefit and group relatedness: Patients in one or the other group may have benefit, but only with this randomized controlled trial we can identify the beneficial operation. As literature is inconclusive according to which stent to use, we do not know the benefits. A drawback for the double J stent is the additional cystoscopy which is needed to remove the stent. There are a few minor complications associated with a cystoscopy such as urinary traction, haematuria or dysuria.

1. INTRODUCTION AND RATIONALE

Kidney transplantation is the only treatment offering long-term benefit to patients with chronic kidney failure. Urological complications after kidney transplantation, such as urinary leakage and ureteral strictures, are associated with significant morbidity, surgical and radiological interventions, prolonged hospital stay and even mortality. The majority of urological complications are related to the ureteroneocystostomy and a first sign is often placement of a percutaneous nephrostomy (PCN) drain(1, 2). It has been demonstrated that stent placement can minimize the number of urological complications (3, 4). Two types of ureteral stents can be used; an internalized double J stent en an externalized single J stent. In our center, we have used an external stent for several years and urological complications are reported up to 9% of the kidney transplant recipients (5). However, in literature the double J stent even has less urological complications. A retrospective study by Vogel et al. including 76 patients with 43 externalized stents and 33 double J stents have reported an incidence of leakage of the ureteroneocystostomy of 13.9% in the externalized stent group compared to 0% in the double J group. Furthermore, they found a 2 day reduction of hospital stay with an internal stent (6). Gomes et al. also retrospectively reviewed the use of external, internal stents and no stent in 2061 kidney transplant recipients. In their cohort, urological complications occurred in 17.3% in the group with external stents, 8.4% in patients that did not receive a stent, and 5.4% in kidney transplant recipients in whom a double J stent was placed (P < .0005)(7). The authors even state: ‘’the use of an external catheter which was associated with a high rate of UC, should be avoided’’. Guleria et al. also reduced their urological complications by changing their technique from a non-stented (7.7%) to a double J stented (for a period of 6 weeks) ureteroneocystostomy (3.8%) (8). Unfortunately, all these studies have a retrospective design and no prospective randomized controlled trials are available. Therefore, in the DUET-trial we will investigate whether double J stenting is indeed superior to the use of an external stent in reducing the number of urological complications after kidney transplantation, as measured by the number of PCN placements.

2. OBJECTIVES

Primary Objective: To assess whether Double J stenting is superior to externalized single J stenting in preventing urological complications after kidney transplantation.

Secondary Objective(s): To assess which kind of stent is superior in reducing the total amount of urological complications, radiological interventions, surgical interventions, haematuria, and urinary tract infections. Stent obstructions or dysfunctions will be scored. Additionally, a quality of life and cost effectiveness analysis will be performed with questionnaires. Validated questionnaires for pain, quality of life, health state, work efforts and disabilities in daily life are measured by VAS, Euro-Qol, SF-36 and ‘Werk en Zorg’. All questionnaires will be filled in pre-operatively and post-operatively at different time points.

3. STUDY DESIGN

This will be a single-centre randomized controlled trial with a superiority design. Randomization by a computerized system will be performed after intubation in the operation room. Physicians participating in this study will be unaware of the randomization sequence thereby guaranteeing concealed allocation. Participants will be included during a period of 3 years, 100 recipients each year. Last follow-up moment of all questionnaires will be after 6 months.

4. STUDY POPULATION

4.1. Population (base)

All adult kidney transplant recipients (>18yrs) are invited to participate. In 3 years 300 recipients will be included.

4.2. Inclusion criteria

All adult kidney transplant recipients in the Erasmus University Medical Center (>18yrs) are invited to participate.

4.3. Exclusion criteria

- Patients with a reconstructed urinary tract or conduit after total or partial cystectomy.
- Patients with bladder dysfunction that requires continuous or intermittent catheterization.
- Patients who do not understand the Dutch language sufficiently to sign the informed consent forms and to fill in the questionnaires
- Donor kidneys with more than one ureter
- Patients with primary FSGS and residual urine production. Because FSGS is known for its quick recurrence in the kidney graft and the first sign is proteinuria. With an externalized stent we are able to distinguish between proteinuria of the transplant kidney and the native kidneys.

4.4. Sample size calculation

This study aims to show that a double J stent results in fewer PCN placements. Based on an two-sided alpha of 0.05, 149 patients are needed to have an 80% power to reject the null hypothesis of no effect when tested with Fisher’s exact test when the double J stent in fact reduces the probability of PCN placement from 9% to 1.5% (3,5) To allow for a few non evaluable cases we randomize 150 patients per arm. (SAS Power and Sample Size 14.1)

5. TREATMENT OF SUBJECTS

5.1. Investigational product/treatment

After the vascular anastomosis the transplant surgeon will perform an extravesical anastomosis as described by Lich-Gregoir (9, 10). A myotomy of 2-3 cm on the anterolateral surface of the bladder dome is performed to expose the mucosa of the bladder wall. A small incision is made in the mucosa. The transplanted ureter is trimmed and spatulated posteriorly. The bladder mucosa is sutured to the ureter with a running absorbable suture. The detrusor muscle is closed over the anastomosis by one or two interrupted absorbable sutures to create a sub-mucosal tunnel with an antireflux mechanism. Participants who are randomized to external stenting will receive an externalised 7 French ureteric stent (Teleflex®). Participants who are randomized to double J stenting will receive a short (12cm) internal Double J 7 French stent(Teleflex®). The tip of both stents will be positioned in the pelvis of the transplanted kidney. The position of the stent will be verified during ultrasonography, which is being performed the day after surgery as standard post-operative care. External stents will be removed 9 days post-operatively. Double J stents will be removed after 3 weeks by cystoscopy in the outpatient clinic of the department of urology. An antibiotic prophylaxis will be used during this procedure based on the latest urinary cultures.

5.2. Use of co-intervention (if applicable)

Recipients will be asked to fill in questionnaires at different time point after transplantation, including a Visual Analogue Score (VAS), quality of life questionnaire (SF-36), Euro-Qol (EQ-5D) and ‘Werk en Zorg’ questionnaires.

5.3. Escape medication (if applicable)

Not applicable

6. INVESTIGATIONAL PRODUCT

6.1. Name and description of investigational product(s)

Single J stent (Teleflex) 7fr

Short (12cm) double J stent (Teleflex) 7fr

Both stents are CE approved and widely used worldwide

6.2. Summary of findings from non-clinical studies

See brochure of Teleflex

6.3. Summary of findings from clinical studies

Not applicable

6.4. Summary of known and potential risks and benefits

Not applicable

6.5. Description and justification of route of administration and dosage

Not applicable

6.6. Dosages, dosage modifications and method of administration

Not applicable

6.7. Preparation and labelling of Investigational Medicinal Product

Not applicable

6.8. Drug accountability

Not applicable

7. NON-INVESTIGATIONAL PRODUCT

This section is not applicable for this study and is therefore left blank.

7.1. Name and description of non-investigational product(s)

7.2. Summary of findings from non-clinical studies

7.3. Summary of findings from clinical studies

7.4. Summary of known and potential risks and benefits

7.5. Description and justification of route of administration and dosage

7.6. Dosages, dosage modifications and method of administration

7.7. Preparation and labelling of Non Investigational Medicinal Product

7.8. Drug accountability

8. METHODS

8.1. Study parameters/endpoints

8.1.1. Main study parameter/endpoint

Percutaneous nephrostomy (PCN) drainages.

8.1.2. Secondary study parameters/endpoints (if applicable)

Urinary tract infection
Haematuria
Radiological interventions
Surgical re-interventions
Stent obstruction or dysfunction
Additionally, a quality of life and cost effectiveness analysis will be performed by using questionnaires. Validated questionnaires for pain, quality of life, health state, work efforts and disabilities in daily life are measured by the VAS, Euro-Qol, SF-36 and ‘Werk en Zorg’.

8.1.3. Other study parameters (if applicable)

Other study parameters are baseline values, which might intervene with the main study parameter: donor age and gender, recipient age and gender, body mass index, smoking, ASA classification, operation time and return to normal daily activities.

8.2. Randomisation, blinding and treatment allocation

This will be a single-centre randomized controlled trial. Randomization will be performed after intubation in the operation room by an electronic system, stratified for type of donor (living/ deceased). Since patients and physicians will notice post operatively the presence of an externalized stent, the study cannot be blinded.

8.3. Study procedures

The work-up for kidney transplantation will be according to the standard protocol. Our “intervention” is internal Double J stenting, since externalized stenting is now standard practice during kidney transplantation.
Post operatively, the double J stent group will have an additional cystoscopy after 3 weeks to remove the stent. Recipients will be asked to fill out a Visual Analogue Score

(VAS), quality of life questionnaire (SF-36), the Euro-Qol (EQ-5D) and the ‘Werk en Zorg’ at different times (Table 1).

Table 1.

Time point	VAS	Euro-Qol	SF-36	Werk en Zorg
Pre-operative	X	X	X	X
Week 2	X	X	X	X
Week 6	X	X	X	X
Month 6	X	X	X	X

- VAS = Visual Analogue Score. Scores the amount of pain and nausea on a scale from none to severe on the basis of numbers (i.e. 0-10).
- EuroQol: Scores the ability to perform daily tasks and measures health outcome.
- SF-36: Scores pain, ability to perform daily tasks and the quality of life.
- Work related questionnaires: score the ability to perform daily tasks.

8.4. Withdrawal of individual subjects

Subjects can leave the study at any time for any reason if they wish to do so without any consequences. The investigator can decide to withdraw a subject from the study for urgent medical reasons.

8.4.1. Specific criteria for withdrawal (if applicable)

Not applicable

8.5. Replacement of individual subjects after withdrawal

Patients will be randomized after intubation, so therefore they will receive the treatment as determined by randomisation. Participants can decide to not fill in the questionnaires anymore. These patients will not be replaced, as we have anticipated on some missing data in our power calculation.

8.6. Follow-up of subjects withdrawn from treatment

Subjects will not be followed up after the study withdrawal, as there are no long term side effects expected. Only patients who insisted to withdrawn from the study who received an internal Double J stent, prior to the removal on 3 weeks after transplantation, will be

stimulated to still have this stent removed after 3 weeks. Prolonged internal stent insertion (> 3 months) has been correlated with stone formations and infections (11) .

8.7. Premature termination of the study

If safety issues arise (according to the DSMB) the study may be ended prematurely

9. SAFETY REPORTING

9.1. Temporary halt for reasons of subject safety

In accordance to section 10, subsection 4, of the WMO, the sponsor will suspend the study if there is sufficient ground that continuation of the study will jeopardise subject health or safety. The sponsor will notify the accredited METC without undue delay of a temporary halt including the reason for such an action. The study will be suspended pending a further positive decision by the accredited METC. The investigator will take care that all subjects are kept informed.

9.2. AEs, SAEs and SUSARs

9.2.1. Adverse events (AEs)

Adverse events are defined as any undesirable experience occurring to a subject during the study, whether or not considered related to [the investigational product / trial procedure/ the experimental intervention]. All adverse events reported spontaneously by the subject or observed by the investigator or his staff will be recorded.

9.2.2. Serious adverse events (SAEs)

A serious adverse event is any untoward medical occurrence or effect that

- results in death;
- is life threatening (at the time of the event);
- requires hospitalisation or prolongation of existing inpatients' hospitalisation;
- requires surgical intervention
- requires PCN placement
- results in persistent or significant disability or incapacity;
- is a congenital anomaly or birth defect; or
- any other important medical event that did not result in any of the outcomes listed above due to medical or surgical intervention but could have been based upon appropriate judgement by the investigator.

An elective hospital admission will not be considered as a serious adverse event.

The investigator will report all SAEs to the sponsor without undue delay after obtaining knowledge of the events, except for the following SAEs: readmissions,

surgical interventions and PCN placements. These events will be reported once a month.

The sponsor will report the SAEs through the web portal *ToetsingOnline* to the accredited METC that approved the protocol, within 7 days of first knowledge for SAEs that result in death or are life threatening followed by a period of maximum of 8 days to complete the initial preliminary report. All other SAEs will be reported within a period of maximum 15 days after the sponsor has first knowledge of the serious adverse events.

9.2.3. Suspected unexpected serious adverse reactions (SUSARs)

Not applicable for this study due to the lack of an investigational medicinal product.

9.3. Annual safety report

In addition to the expedited reporting of SAEs, the sponsor will submit, once a year throughout the clinical trial, a safety report to the accredited METC, competent authority, and competent authorities of the concerned Member States.

This safety report consists of:

- a list of all suspected (unexpected or expected) serious adverse reactions, along with an aggregated summary table of all reported serious adverse reactions, ordered by organ system, per study;
- a report concerning the safety of the subjects, consisting of a complete safety analysis and an evaluation of the balance between the efficacy and the harmfulness of the medicine under investigation.

9.4. Follow-up of adverse events

All AEs will be followed until they have abated, or until a stable situation has been reached. Depending on the event, follow up may require additional tests or medical procedures as indicated, and/or referral to the general physician or a medical specialist. SAEs need to be reported till end of study within the Netherlands, as defined in the protocol

9.5. [Data Safety Monitoring Board (DSMB) / Safety Committee]

A Data Safety Monitoring Board will be installed to evaluate this study after every 50 included patients. The following people will take place in this Board:

Prof. Dr. H.J. Metselaar, department of gastroenterology and hepatology

Drs. S.P. Willemsen, department of biostatistics

Dr. R.W.F. de Bruin, department of surgery

The advice(s) of the DSMB will only be sent to the sponsor of the study. Should the sponsor decide not to fully implement the advice of the DSMB, the sponsor will send the advice to the reviewing METC, including a note to substantiate why (part of) the advice of the DSMB will not be followed.

10. STATISTICAL ANALYSIS

10.1. Primary study parameter(s)

The primary study parameter is PCN placement within six months after transplantation. We will present this outcome in both arms both as a number and as a percentage. We will test is the probability of PCN placement is equal by means of Fisher's Exact test. A p-value below 0.05 (two sided) will be considered as statistically significant. All patients will be analyzed by the intention-to-treat approach.

10.2. Secondary study parameter(s)

The following outcomes will be studied using a linear mixed model:

- The level of creatinin and eGFR after transplantation
- The answers to the SF-36 questionnaire
- The answers to the EuroQol questionnaire
- The answers to the work related questionnaire

As predictors we use indicator variables for the different time points and an interaction of each of the follow-up time points with the treatment arm. Note that at baseline we see both arms of the trial as a single group.

For each response we choose the most suitable covariance structure from the compound Symmetry, compound symmetry heteroskedastic autoregressive, autoregressive Heteroskedastic, Toeplitz and heteroskedastic Toeplitz based on the Akaike information criteria. Nonsignificant terms will not be removed from the model. The primary contrast we test is whether the profiles of the arms overlap. When the result of this multivariate test is significant we will also test the results at the various follow-up time points. Note that we will not apply a correction for multiple testing looking at multiple responses.

The following categorical outcomes will be compared between the treatment arms by means of a continuity corrected Chi square test:

- Hematuria
- Urinary tract infection
- Urosepsis
- Reoperation
- Radiologic interventions
- Tacrolimus toxicity
- Kidney replacement therapy
- Rejection
- Graft failure
-

When any cell is empty or when more than 20% of the expected counts are less than five Fishers exact test will be performed for this outcome instead of the Chi square test.

The following continuous outcomes will be compared between the treatment arms by means of a Mann-Whitney U test:

- First and second warm ischemia time
- Cold ischemia time
- Time needed to make an urether-bladder anastomosis
- Blood loss during transplantation
- Duration of transplantation
- Duration of admission

All tests will be performed in a two-sided fashion with an alpha of 0.05. No multiplicity adjustment will be made. We will present the baseline characteristics of both treatment arms by showing the median and IQR for continuous variables and numbers and percentages for categorical variables. No statistical test will be performed on these values.

The cost effectiveness of the double J stent will be calculated as followed:

$$\frac{Costs\ Double\ J\ stent - Costs\ Splint}{Percentage\ of\ PCN\ placements\ in\ Double\ J\ stent\ arm - Percentage\ of\ PCN\ placements\ in\ Splint\ arm}$$

The costs of the double J stent will include the costs of the cystoscopy in the outpatient clinic for the removal of the double J stent.

10.3. Other study parameters

Not applicable

10.4. Interim analysis (if applicable)

No interim analysis will be performed.

11. ETHICAL CONSIDERATIONS

11.1. Regulation statement

The study will be conducted according to the principles of the Declaration of Helsinki (59th WMA General Assembly, Seoul, October 2008) and in accordance with the Medical Research Involving Human Subjects Act (WMO).

11.2. Recruitment and consent

The recruitment will start when a favourable opinion by the accredited METC has been given to conduct the study. During the surgical outpatient visit, patients receiving a kidney of a living donor will be informed and asked for their cooperation by the investigator. This will be different in patients receiving a graft of a deceased donor. Patients who are on the Dutch transplant waiting list for a deceased donor, will not receive the information about the study during their surgical outpatient visit as the average waiting time on the waiting list is currently 4 years. The study will probably be finished at time they will receive a kidney transplant. The timing of a deceased kidney transplant is always unexpected and very often during after-hours. Therefore these patients will be informed and asked for their cooperation pre-operatively by the operating surgeon. These patients have time to consider participation until 2 hours before the operation. Informed consent will be signed before the operation.

11.3. Objection by minors or incapacitated subjects (if applicable)

Not applicable

11.4. Benefits and risks assessment, group relatedness

There is no additional risk for the patient or the operation. Patients in one or the other group may have benefit, but only with this randomized controlled trial we can identify the beneficial operation. As literature is inconclusive to which stent to use, we do not know the benefits. A drawback for the double J stent is the additional cystoscopy which is needed for stent removal. There are a few minor complications associated with a cystoscopy such as urinary traction, haematuria or dysuria.

11.5. Compensation for injury

The sponsor (also) has an insurance which is in accordance with the legal requirements in the Netherlands (Article 7 WMO). This insurance provides cover for damage to research subjects through injury or death caused by the study.
The insurance applies to the damage that becomes apparent during the study or within 4 years after the end of the study.

11.6. Incentives (if applicable)

There will be no compensation for participation in this study.

12. ADMINISTRATIVE ASPECTS, MONITORING AND PUBLICATION

12.1. Handling and storage of data and documents

The coordinating investigator collects all data. Every patient is coded into numbers. The coordinating and principal investigators have access to the source data. Data will be stored for 15 years.

12.2. Monitoring and Quality Assurance

There is an intermediate risk for this study. We will use the Erasmus Medical Center Monitor Plan B for this study.

12.3. Amendments

Amendments are changes made to the research after a favourable opinion by the accredited METC has been given. All amendments will be notified to the METC that gave a favourable opinion. All substantial amendments will be notified to the METC and to the competent authority. Non-substantial amendments will not be notified to the accredited METC and the competent authority, but will be recorded and filed by the sponsor.

12.4. Annual progress report

The sponsor/investigator will submit a summary of the progress of the trial to the accredited METC once a year. Information will be provided on the date of inclusion of the first subject, numbers of subjects included and numbers of subjects that have completed the trial, serious adverse events/ serious adverse reactions, other problems, and amendments.

12.5. Temporary halt and (prematurely) end of study report

The investigator/sponsor will notify the accredited METC of the end of the study within a period of 8 weeks. The end of the study is defined as the last patient’s last visit.
The sponsor will notify the METC immediately of a temporary halt of the study, including the reason of such an action. In case the study is ended prematurely, the sponsor will notify the accredited METC within 15 days, including the reasons for the premature termination. Within one year after the end of the study, the investigator/sponsor will submit a final study report with the results of the study, including any publications/abstracts of the study, to the accredited METC.

12.6. Public disclosure and publication policy

At the end of the study the research data may be published, investigators should be mentioned in the article.

13. STRUCTURED RISK ANALYSIS

13.1. Potential issues of concern

Stent placement is a very know phenomena and is broadly used in both urological and surgical operations. In this study we hypothesize that double J stent placement is superior in minimizing the number of urological complications, as double J stents have a retrospectively reported incidence of 0-5.4% (6-8) and our data with externalized single J stents report an incidence of 9-20% (5, 12) urological complications. We have considered the potential risk of the additional risk of the cystoscopy but we have found these risks acceptable and therefore included the double J stent in this protocol. The stents are both CE approved and are widely used worldwide.

13.2. Synthesis

Reducing the number of urological complications after kidney transplantation has a high priority. If we were able to reduce the number of urological complications from 20% to 5% with a double J, would justify the additional cystoscopy and its (rare) complications. The cystoscopy will be performed by the department of urology under local anesthesia with an antibiotic prophylaxis (based on latest urinary culture) in an outpatient visit, therefore minimizing the additional risks of this cystoscopy.

14. REFERENCES

1. Dols LF, Terkivatan T, Kok NF, Tran TC, Weimar W, JN IJ, et al. Use of stenting in living donor kidney transplantation: does it reduce vesicoureteral complications? Transplant Proc. 2011;43(5):1623-6.
2. Miraglia R, Caruso S, Milazzo M, Salis P, Luca A, Gridelli B. Efficacy of interventional radiology procedures for the treatment of early ureteral complications after kidney transplantation. Transplant Proc. 2006;38(9):2919-20.
3. Wilson CH, Rix DA, Manas DM. Routine intraoperative ureteric stenting for kidney transplant recipients. Cochrane Database Syst Rev. 2013;6:CD004925.
4. Mongha R, Kumar A. Transplant ureter should be stented routinely. Indian J Urol. 2010;26(3):450-3.
5. Ooms LS, Spaans LG, Betjes MG, Ijzermans JN, terkivatan T. Minimizing the number of urological complications after kidney transplantation: A comparative study of two types of external ureteral stents. Exp Clin Transplant. 2016.
6. Vogel T, Utech M, Schmidt F, Holscher Keplin W, Diller R, Brockmann J, et al. Double-J Versus External Ureteral Stents in Kidney Transplantation: A Retrospective Analysis. Nephrourol Mon. 2015;7(4):e27820.
7. Gomes G, Nunes P, Castelo D, Parada B, Patrao R, Bastos C, et al. Ureteric stent in renal transplantation. Transplant Proc. 2013;45(3):1099-101.
8. Guleria S, Chahal R, Madaan S, Irving HC, Newstead CG, Pollard SG, et al. Ureteric complications of renal transplantation: the impact of the double J stent and the anterior extravesical ureteroneocystostomy. Transplant Proc. 2005;37(2):1054-6.
9. Gregoir W. [the Surgical Treatment of Congenital Vesico-Ureteral Reflux]
Le Traitement Chirurgical Du Reflux V'esico-Ur'et'eral Cong'enital. Acta Chir Belg. 1964;63:431-9.
10. Gregoir W, Vanregemorter G. [Congenital Vesico-Ureteral Reflux]
Le Reflux V'esico-Ur'et'eral Cong'enital. Urol Int. 1964;18:122-36.
11. Adanur S, Ozkaya F. Challenges in treatment and diagnosis of forgotten/encrusted double-J ureteral stents: the largest single-center experience. Ren Fail. 2016;38(6):920-6.
12. Slagt IK, Dor FJ, Tran TC, Kimenai HJ, Weimar W, Ijzermans JN, et al. A randomized controlled trial comparing intravesical to extravesical ureteroneocystostomy in living donor kidney transplantation recipients. Kidney Int. 2014;85(2):471-7.
